# Women’s adoption of a web-based intervention for stress urinary incontinence: a qualitative study

**DOI:** 10.1186/s12913-021-06585-z

**Published:** 2021-06-12

**Authors:** Lotte Firet, Theodora Alberta Maria Teunissen, Rudolf Bertijn Kool, Lukas van Doorn, Manal Aourag, Antoinette Leonarda Maria Lagro-Janssen, Willem Jan Jozef Assendelft

**Affiliations:** 1 Department of Primary and Community Care, Radboud Institute for Health Sciences, Radboud University Medical Center, Geert Grooteplein 21, Postbox 9101, 6500 Nijmegen, HB Netherlands; 2 IQ Healthcare, Radboud Institute for Health Sciences, Radboud University Medical Center, Nijmegen, Netherlands

**Keywords:** E-health, Urinary incontinence, Women, Adoption, Implementation research, FITT framework, Qualitative study, Pelvic floor muscle training

## Abstract

**Background:**

Stress urinary incontinence (SUI) is common among women and affects their quality of life. Pelvic floor muscle training is an effective conservative therapy, but only a minority of women seek help. E-health with pelvic floor muscle training is effective and increases access to care. To implement an e-Health intervention in a sustainable way, however, we need to understand what determines adoption. The aim is to investigate the barriers and facilitators to adopting an e-Health intervention among Dutch women with stress urinary incontinence.

**Methods:**

Semi-structured telephonic interviews were carried out among participants of the Dutch e-Health intervention for women with stress urinary incontinence. Women were purposively sampled. The ‘Fit between Individuals, Task and Technology’ (FITT) framework was used for both the data collection and data analysis, to gain a more in-depth insight into the adoption of the intervention.

**Results:**

Twenty women were interviewed, mean age 51 years and mostly highly educated. The adoption of e-Health for women with SUI mainly depends on the interaction between users and e-Health, and users and pelvic floor muscle training exercises. Facilitators for the adoption were the preference for an accessible self-management intervention, having a strong sense of self-discipline and having the ability to schedule the exercises routinely. Women needed to possess self-efficacy to do this intervention independently. Barriers to the adoption of e-Health were personal circumstances restricting time for scheduling pelvic floor muscle training and lacking skills to perform the exercises correctly. Despite guidance by technical features several women remained uncertain about their performance of the exercises and, therefore, wanted additional contact with a professional.

**Conclusions:**

For stress urinary incontinence e-Health is an appropriate option for a target audience. Use of the FITT framework clearly demonstrates the conditions for optimal adoption. For a subgroup it was a suitable alternative for medical care in person. For others it identified the need for further support by a health care professional. This support could be provided by improvements of technical features and incorporating modes for digital communication. The additional value of integration of the e-Health intervention in primary care might be a logical next step.

**Trial registration:**

The study was prospectively registered in the Netherlands Trial Registry (NTR) NTR6956.

**Supplementary Information:**

The online version contains supplementary material available at 10.1186/s12913-021-06585-z.

## Background

Urinary incontinence is a highly prevalent condition with a negative impact on quality of life [1, 2]. Stress urinary incontinence (SUI) is the most common subtype, which mainly affects women. It is defined as ‘the complaint of involuntary urinary leakage on effort or exertion, or on sneezing or coughing’ [3]. A recent Dutch study showed that one in four women have SUI [4], with the majority being between 40 and 59 years of age [5]. Although SUI is not a life-threatening disease, it is a serious burden on quality of life and well-being. A systematic review showed that urinary incontinence affects quality of life on both psychological and physical aspects [6]. Women with SUI encounter a range of emotions: problems with intimate relations, depression, embarrassment, low self-esteem and even suicide [2]. SUI typically limits women in daily activities, such as exercises, or activities with relatives, as they could provoke leakage [1].

General practitioners (GPs) only see a tip of the iceberg of their female patients with SUI because only one in three women seek help for their complaint [7, 8]. This low consultation rate is not only because women feel shame, regard SUI as a normal process of ageing or of giving birth, but also because that they think there is no treatment available [9, 10]. However, there are effective treatment options for SUI available, such as pelvic floor muscle treatment (PFMT), which can be guided by GPs, nurse practitioners or pelvic floor physiotherapists [11]. Most GPs prefer to refer women to a specialized physiotherapist, because they lack time and knowledge to guide this therapy properly [12, 13]. GPs acknowledge that care for women with SUI can still be improved [13].

E-Health could be a solution to improve care for women with SUI. Interviews with Dutch GPs by our group showed that GPs consider the use of e-Health with PFMT as a welcome new tool for treating women with SUI [14]. When interviewed, Dutch women with SUI reported that they expected that e-Health could serve as a tool to bridge obstacles they encountered in regular care, but they underlined they felt the need to also be able to meet with a person during therapy for SUI [15].

Two Swedish RCTs showed that Internet-based or app-based treatment with PFMT was effective in reducing urinary leakage and improving incontinence-related quality of life [16, 17]. In addition, in qualitative studies alongside, both interventions were positively evaluated by their participants [18, 19]. Users felt empowered because there was a treatment option for their problem. Women appreciated the remote contact they had during an internet-based intervention, because they felt that they were not alone, without the need to disclose themselves face-to-face [18]. The results from these Swedish studies are in line with a qualitative study among Dutch women with urinary incontinence who used a mobile app [20] .

These studies evaluated women’s experiences with digital therapy and their satisfaction in general. However, a more systematic, in-depth analysis of which factors determine the actual usage of e-Health is missing and is necessary to implement an e-Health intervention in a sustainable way. Adoption is the technical term that defines the usage of a new technology and it is one of the stages that need to be addresses during an implementation process [21, 22]. Studying adoption would provide information about reasons for using, tend to stop using, or stop using an intervention for women with SUI. This information will guide health care providers and policymakers in implementing an e-Health intervention for SUI. The aim of this study is to investigate the barriers and facilitators to the adoption of an e-Health, web-based, intervention for SUI among Dutch women.

## Methods

### Study design

This study is part of a research project on the implementation of an internet-based intervention in the Netherlands [23]. In this qualitative study, semi-structured interviews were conducted among Dutch women who participated in this e-Health intervention for SUI.

### Participants

All women interviewed in this study took part in the ‘*Baas over je blaas’* [24] (‘*Master your own bladder*’) e-Health intervention, which was the subject of an implementation study*.* The methods of this study have been described previously [23]. In short, it concerns a web-based intervention in which PFMT is addressed in eight escalating steps (modules), with each step opening after the woman has completed a training report. Each step contains background information, a training program and a test exercise that enables women to check whether they gained the correct skills. ‘*Baas over je blaas’* is based on the Swedish internet-based intervention named ‘*Tät-treatment of Stress Urinary Incontinence’,* which was the subject of an RCT [16]. The eContinence group from Umeå university translated the program to Dutch and gave permission to use the program for research purposes through a non-commercial license agreement. Our research group fully adapted the eHealth intervention and only added audio recordings in which the text was read aloud. Between July 2018 and March 2019, women with SUI > 18 years old could sign up for this study without referral from a healthcare professional. They first had to provide informed consent and complete an online questionnaire that enabled the researcher to check their eligibility. Selected women had 3 months access to the intervention, and they received automated e-mail reminders to stimulate adherence to the training program during this period. They could do the training program at their own pace, and they could contact the researcher, who is also a GP in training (LF), for questions by e-mail.

In the baseline questionnaire, women were asked if they were willing to participate in an interview after using the online training program. To minimize recall bias, we aimed to conduct the interviews within 6 months after access to the intervention was closed and we succeeded to interview the majority within 4 months (mean 3.7 months). Forty-three women were contacted by e-mail by LF to ask if they agreed to be interviewed by a medical student (LvD, MA). Twenty women agreed and were contacted by the student via e-mail or phone to make an appointment for the interview. We strived to include women with variation in age, education level, symptom severity and adherence to the intervention.

### Conceptual framework

**Fig. 1:**
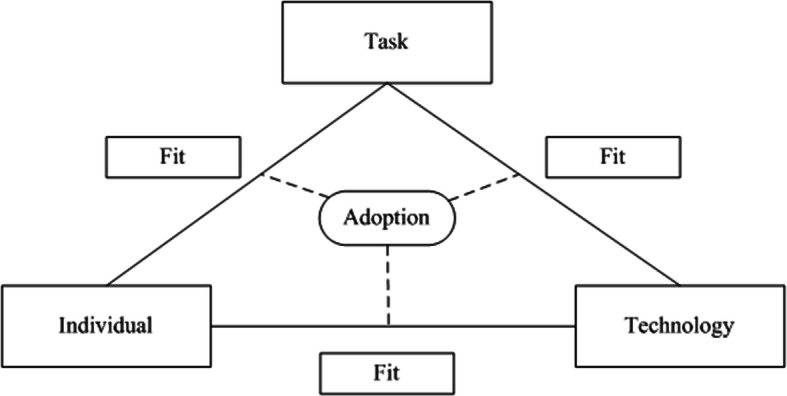
FITT framework as created by Ammenwerth et al. [25]

During our process of data collection and data analysis we used the Fit between Individuals, Task and Technology (FITT) framework created by Ammenwerth et al. [25]. We choose this framework because it focusses on adoption specifically, it encompasses a broad range of factors that determine adoption, and it was designed for the health care sector. The rationale is that adoption of a new technology depends on the interaction, the fit, between individual-, technology- and task-related attributes. According to this framework we investigated the fit between attributes of individuals (e.g., personal circumstances and computer skills), of attributes of technology (e.g., content and access) and attributes of tasks (e.g., adherence and scheduling) (Fig. 1). Interaction between individuals and technology in this study comprised, for example, that women should have the computer skills required to handle the e-Health intervention. Interaction between individuals and tasks included that women with SUI needed to be able to schedule PFMT exercises in their daily lives. The interaction between tasks and technology, lastly, comprised that the e-Health intervention had to support PFMT performance by providing clear content that clarified the task.

### Data collection

Telephonic interviews were held between July and October 2019 by two trained medical students, one male and one female. Both interviewers had no relation to the participants. Interviews took between 30 and 40 min on average. The FITT framework was leading in the development of the interview guide (see Additional file 1) because the attributes of the three components (individuals, task and technology) needed to be discussed during the interviews to investigate the interaction between them. The input on the interview guide of these three components was based on the participants’ previous responses to open-ended questions, which they filled in during their participation in the e-Health intervention. These questions asked women about their positive and negative experiences with the e-Health intervention and the answers were analyzed thematically. Furthermore, we used input for the interview guide from the literature about known PFMT adherence modifiers [26] and about internet-based treatment for SUI [18], added with expertise from the authors. Prior to the interview with the first participant, the interview guide was pilot tested twice with women who were simulated patients and who received anonymous case vignettes of eHealth participants. No major amendments were made after pilot testing.

Semi-structured interviews were conducted, meaning that women were asked predetermined open-ended questions about key topics in the interview guide. Follow-up questions emerged from the dialogue and were not determined prior to the interview [27]. Key topics that were addressed were: reasons for participation, previous experiences with treatment for SUI, expectations prior to the start of the intervention, effects of the intervention, performing and prioritizing PFMT in daily life, adherence and technical aspects of the intervention. We explicitly asked women about their experiences with unsupervised treatment, because literature shows that women feel the need to meet with someone during the training program [15]. After seven interviews, we adjusted the interview guide [28]. We explicitly asked women, who said that they missed personal contact during the intervention, how in their view e-Health and contact could be best combined. Furthermore, we wanted to gain more in-depth information on how women performed PFMT in daily life by focusing on motivation, planning, prioritizing and a women’s daily context.

### Data analysis

Interviews were audio-recorded and transcribed verbatim anonymously. The interviewer conducted member checking by summarizing the input from the participants at the end of the interview. Data saturation was reached after 19 interviews, as no new codes emerged. We conducted a last interview because that appointment had already been set, and this interview did indeed reveal no new results. The researchers repeatedly read the transcripts to familiarize themselves with the data.

The transcribed interviews were analyzed using the ATLAS.ti version 8 program using thematic analysis. First, open-coding was applied to the interview fragments. Two researchers coded independently (LvD and MA), and they compared their codes after every three interviews. In case of disagreement, a third researcher (LF) gave her opinion. These codes were also labelled deductively with one of the three components of the FITT framework (individuals, task or technology). When codes could not be clearly assigned to one of these components, they were discussed by the supervising committee. Codes were gradually merged into categories. After categorizing, we checked for individuals-technology, individuals-task and task-technology interaction. The category ‘*experience from previous treatment*’, for example, included both individuals-related and task-related codes, and we concluded, therefore, that this category enhances interaction between the individuals and the task (Table 1).

**Table 1:** Example of analyzing process illustrating the interaction between individuals and task

Code names	FITT Label	Category	Theme within interaction
• Previous treatment (PFMT) did not help.	Individuals	Experience from previous treatment	Skills / experience with PFMT - complexity
• Visit to pelvic floor physiotherapist prior to e-Health intervention clarified the exercise.	Task		

The categories contained codes that were either barriers or facilitators to the adoption of e-Health. All categories that indicated an interaction between individuals and task were clustered, as were all categories indicating interaction between individuals and technology and between task and technology. Within these interactions, the categories that belonged to that interaction provided a thematic description (see Additional file 2). The interactions were leading for determination of the final results because, taken together, they determined adoption.

Quotes are used to illustrate the main findings, and these quotes are followed by identifier number, age, incontinence severity according to the sumscore on the International Consultation on Incontinence Questionnaire Short Form (slight, moderate, severe or very severe) and adherence level (reaching steps 1–8). To indicate the number of participants who shared an idea on one topic, we used the words *few, several, many, most* and *all,* reflecting a number of 1–4, 5–9, 10–14, 15–19 or 20 participants, respectively. The Consolidated Criteria for Reporting Qualitative Research (COREQ) were used to report the results [29] (see Additional file 3).

## Results

**Fig. 2:**
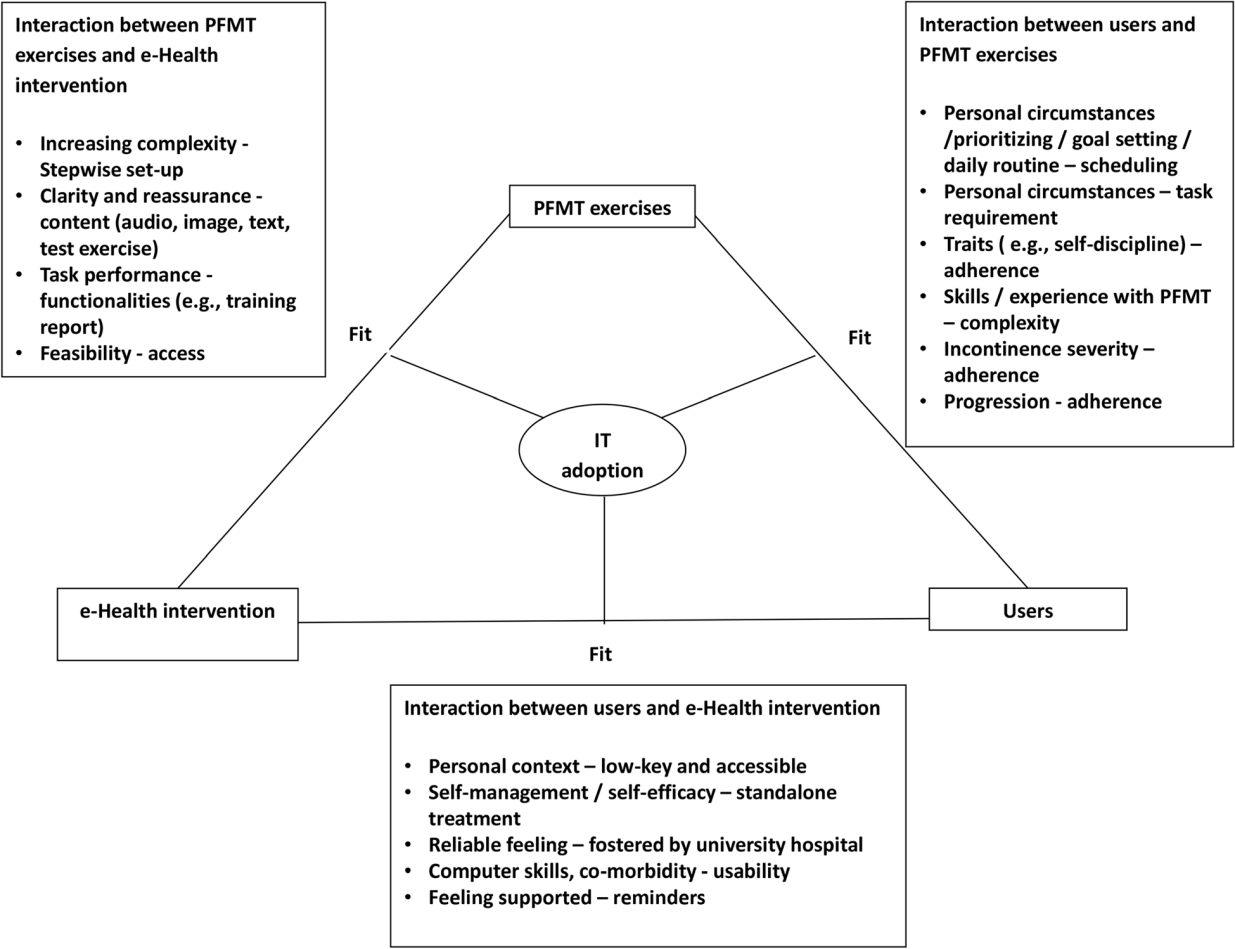
The adoption of the eHealth intervention determined by the fit between users, eHealth intervention and PFMT

Twenty women were interviewed; their mean age was 51 years old, and the majority of them had a moderate degree of incontinence severity. Twelve women were highly educated, meaning Master’s until doctoral degree. Almost half the women had never visited a healthcare professional for their urinary incontinence, whereas the other half had visited a GP or a pelvic floor muscle therapist (Table 2). Previous treatment had either not been effective or had taken place too long ago, with obtained results having decreased. The adoption of e-Health as studied by the FITT framework is presented according to the interactions within the FITT framework (Fig. 2). In the paragraph headings, the words “users”, “PFMT exercises” and “e-Health” correspond to the words “individuals”, “task” and “technology” from the FITT framework. For each paragraph quotes from the interviews are displayed in Tables 3, 4 and 5 respectively.

**Table 2:** Demographic characteristics of 20 participants

Variables	Range	n (%)
Age in years	< 30	1 (5%)
	31–40	3 (15%)
	41–50	5 (25%)
	51–60	7 (35%)
	61–70	2 (10%)
	> 70	2 (10%)
Education level ^a^	Low	3 (15%)
	Intermediate	5 (25%)
	High	12 (60%)
Symptom severity ^b^	Slight	1 (5%)
	Moderate	14 (70%)
	Severe	5 (25%)
	Very severe	0 (0%)
Level of adherence ^c^	Step 1–5	10 (50%)
	Step 6–8	10 (50%)
Previous consultation or therapy	None	9
	General practitioner	3
	Pelvic floor physiotherapist	8

^a^ Education level divided into: low = primary school until upper secondary; intermediate = post-secondary non-tertiary until Bachelor’s; high = Master’s until doctoral

^b^ Total sum score on the International Consultation on Incontinence Questionnaire Short Form (ICIQ-UI SF) [30]

^c^ Adherence to the web-based intervention based on the highest step reached, with a maximum of eight steps

**Table 3:** Quotes corresponding with ‘interaction between users and e-Health’

Quotes ‘interaction between users and e-Health’	
Q1	*“The advantage of the website is that you can do it in your own time and pace, I consider that as a great advantage. You do not have to plan an appointment.”* (ID 10411, 49 years, moderate, step 8)
Q2	*“Participant: I thought where could I possibly plan this appointment cause I have a child with a handicap, so my days are scheduled in other ways than those of other people. […*] *So I thought where would I find the time, just can’t do it.”* (ID 10432, 43 years, moderate, step 1)
Q3	*“I just wanted to see if it is a remedy with a very easy approach […] I try to avoid the medical system as much as possible.”* (ID 10262, 53 years, moderate, step 7)
Q4	*“I thought I’d start trying to fix this problem through the internet. It is actually similar to a visit with the physiotherapist, when I went there for my shoulder I ended up doing certain exercises independently, without anyone’s help.”* (ID 10363, 48 years, moderate, step 8)
Q5	*“The background of the website is quiet and not overloaded with colors and text, so it is easy to read, because longer text fragments usually give me trouble.”* (ID 10432, 43 years, moderate, step 1)
Q6	*“I consider it as an advantage that you can solve this problem yourself, you do not really need help, you only have to follow the steps in this program.”* (ID 10326, 71 years, moderate, step 8)
Q7	*“I would have liked it if, just briefly, they would have asked some questions, such as ‘how does it go?’, ‘how are you performing in this regard?’ and ‘how do you feel when doing this?’, which helps you finding out if something goes wrong.”* (ID 10420, 32 years, severe, step 2)
Q8	*“I did not miss face-to-face contact at all […*], *but what I liked about the program was that they ask you every week ‘where are you in the program?’, ‘how are you doing?’. […] It feels like someone is helping you along, actually. That was really like coaching, like someone who is really involved. I thought that was a real strength.”* (ID 10272, 65 years, severe, step 8)
Q9	*“For me it would have worked if I had had one telephone consultation a month […] whether in person or by telephone […] If you know ‘someone is going to ask me’, this helps you want to achieve something.”* (ID 10265, 45 years, severe, step 2).

**Table 4:** Quotes corresponding with ‘interaction between users and PFMT exercises’

Quotes ‘interaction between users and PFMT exercises’	
Q1	*“I just do it whenever I get off the train. It’s like a Pavlov reaction, which might sound a bit strange, but it makes it very easy. I don’t need to think about it because I’m doing it again and again”* (ID 10262, 53 years, moderate, step 7)
Q2	*“I started training again in my holidays, because this way I felt ‘I would not have an excuse for not performing those exercises of ‘Baas over je blaas’ three times a day.”* (ID 10330, 57 years, moderate, step 5)
Q3	“Well, if you have a two-year old child and mummy is lying down, then she will start jumping up and down or asking for attention. She just won’t accept that you lie down *laughing*; and so you can’t take a moment’s rest to do it.” (ID 10435, 36 years, slight, step 2)
Q4	“*Sometimes I only practiced twice a day, because it is hard to combine it with your daily duties. For example, how would my colleagues react if I would lie down on the floor during work?”* (ID 10434, 60 years, moderate, step 7)
Q5	“*… but it’s in my nature. Once I start something, then I am a go-getter.”* (ID 10326, 71 years, moderate, step 8)
Q6	“During the first contractions, you’re thinking ‘well, I think I’m doing alright’. But then at a certain point, you’re thinking: ‘I guess I’m contracting my buttocks, or my legs, and I don’t feel anything happening inside’. *Giggling* Well, yeah I really doubted if I was doing the right thing.” (ID 10285, 23 years, moderate, step 3)
Q7	*“To me everything was clear because I’d seen a physiotherapist, but without that experience, you know, it is quite an area down there. Then I would think: ‘How should you contract, should you contract in front, should you pull everything inside, how does it work with your rectum, that sort of thing.’”* (ID 10292, 58 years, severe, step 2)
Q8	*“The reason why I started was my incontincence during a sportclass, which I rarely do, so it did no bother me that much on the long run. Because I was not hindered often, my motivation decreased.”* (ID 10435, 36 years, slight, step 2)
Q9	*“You feel much better if you don’t have to think about wearing an incontinence pad, or needing to go to the toilet right now, or stuff like that. So that’s what really motivated me, and to be honest I had not expected things to improve this much. So that motivated me to do it.”* (ID 10426, 53 years, moderate, step 5)

**Table 5:** Quotes corresponding with ‘interaction between PFMT exercises and e-Health’

Quotes ‘interaction between PFMT exercises and e-Health’	
Q1	*“You know you cannot start with the toughest exercises and you don’t know which exercises you should do to gain the best result. I liked the fact that you progressed step-by-step.”* (ID 10369, 49 years, moderate, step 6)
Q2	*“Quite soon I noticed my muscles became stronger and that is because you start on a low level. In the beginning it was impossible to be hard on myself, because after three seconds contracting, my muscle loosened up. But every next time I thought, I can do this and I could reach a higher level. I really felt the setup with the different levels was very good.”* (ID 10272, 65 years, severe, step 8)
Q3	*“Interviewer: what was the reason for keeping up your motivation?* *Interviewee: I enjoyed it because I was curious towards the new exercises. I think that was a good method, not seeing the whole program from the start. Every time you get something new, making you sort of curious to what is coming.”* (ID 10236, 56 years, moderate, step 8)
Q4	*“What I mean by a video is, for instance, that you have someone explain it and then show an image of how it’s done, or perhaps someone who demonstrates how to do it, with different lying positions, something like that.”* (ID 10285, 23 years, moderate, step 3)
Q5	*“At first […] it was hard to discover those muscles. But every time I was reassured by the program: ‘You will be alright, perhaps not immediately but you will soon enough.’ And they were right.”* (ID 10326, 71 years, moderate, step 8)
Q6	*“At first, I thought am I doing this right? […] But I gradually discovered that I improved, even with the tougher and longer exercises, and that gave me a lot of strength. My own progress gave me the confidence that I was doing it right.”* (ID 10363, 48 years, moderate, step 8)
Q7	*“I could have continued with steps 2 and 3 much sooner, because I knew how to do it. But I felt it would be pointless to do things too quickly, what would be the benefit of that? […] Even though I can do it in five minutes, it could still be the case that my muscles are not functioning well enough. So staying in one step until I felt I truly mastered it worked well for me.”* (ID 10432, 43 years, moderate, step 1)
Q8	*“I had to calculate and measure the length of the exercises, I found that complicated. I also use a women fitness app for example, which is super easy because it is counting down.”* (ID 10285, 23 years, moderate, step 3)
Q9	*“It was a pity that the programme finished, because I remained a bit longer in each step to be sure that I could go on to the next step. Due to this, I was not able to get to step 8.”* (ID 10426, 53 years, moderate, step 5)

### Interaction between users and e-health

Most women mentioned that they participated in the e-Health intervention because it was an option that was easy to access for treating their urinary incontinence. Several women said *“it does not hurt to try”*. Women said it was easy to access because they appreciated to participate anonymously and free of charge. Furthermore, they preferred not having to make face-to-face appointments, as well as being able to perform the exercises in their own time and at their own pace (Q1). The accessibility of e-Health made it fit well into the women’s personal context, as was mentioned by a few women. One woman said that her circumstances were suitable for participation in this e-Health intervention as she was awaiting knee surgery and wished to tackle her incontinence problem in the meantime because it was always bothering her during tennis. Another woman said that being a caregiver left her with little time to visit a healthcare professional (Q2).

The wish for self-managing urinary incontinence was another facilitator for starting with e-Health. Several women liked the idea of trying e-Health as a first step before consulting their doctor, or even wanted to bypass the medical system or surgery altogether (Q3). They possessed a certain degree of self-efficacy that made them aware that exercises were needed to tackle this problem and believed they were able to do them without the help of a professional (Q4). Still, the fact that the website had been set up by an academic hospital made a few women feel it was reliable.

The usability of the website showed a clear interaction between users and e-Health. Most women were satisfied with its use, because it was easy to navigate through the website, and it was compatible with a computer or tablet. The audio-function and the simple layout were facilitators for a few women with reading problems due to co-morbidities (Q5). The high information density and login problems were barriers to using the website, which was stated by several women. One woman lacked computer skills and had her son print the exercises, because she could not use the website herself.

An often-mentioned topic was the standalone aspect of e-Health, that is, the absence of face-to-face or telephone contact with a professional. This was regarded either as a facilitator or as a barrier for using the website. Half the women felt that the absence of contact enabled them to solve this problem themselves. All you had to do is follow the eHealth program, they did not need or expect contact (Q6). One woman said that for more intensive contact she would need to consult a physiotherapist, but she consciously opted for e-Health. The possibility to ask questions by e-mail was regarded as being sufficiently supportive. In contrast, half the other women regarded the absence of personal contact as a barrier for using the website as they could not regularly ask questions or receive feedback on their performance (Q7) To a few women this feedback also enhanced a vaginal examination by a professional, to learn whether they contract their pelvic floor muscles correctly. One woman said that she felt lonely when using the website because she struggled with how to perform the exercises. Women also regarded diagnostic self-assessment as a disadvantage. Two women, consequently, consulted a healthcare professional during the training program and discovered that they did not have SUI but an overactive pelvic floor, and they were advised to discontinue the intervention.

E-Health without personal contact was considered by several women as non-committal because they missed encouragement and they did not have the feeling they were doing it for anyone. However, several women mentioned that the weekly e-mail reminders facilitated them to keep using the intervention and made them feel that someone kept an eye on them (Q8). For others, e-mail reminders were barriers, because they overloaded their mailbox or created a sense of guilt as these women then realized they had not practiced in the previous period.

Women provided different suggestions for combining the e-Health intervention with personal contact. For the majority, remote contact via telephone, chat or videocall would be sufficient to meet their need for asking questions (Q9). Others preferred face-to-face contact as this would feel more personal to them and enable them to receive feedback by vaginal examination. Peer contact was also mentioned. Contact could take place either on request or at a pre-scheduled time, which was recommended by women who went to see a physiotherapist previously. Except for those women who wished to have a vaginal examination, the background of the person to provide the contact with does not matter, as long as he or she was sincerely interested in them, had time and had knowledge of the topic.

### Interaction between users and PFMT exercises

The interaction between women and the performance of PFMT exercises was strongly determined by their ability to fit these into their everyday lives. Women said that integrating the exercises into their daily routine or to set personal goals facilitated them to train on a regular basis. They recommended chosing specific moments for integrating the exercises, such as when waiting in line in the supermarket or when travelling to work (Q1). Making a habit of doing the exercises in their daily lives gave them the idea that the exercises were not time-consuming. A few women also noticed that changes in daily routine, such as holidays or retirement, could either facilitate or impede adherence to the schedule (Q2).

Women used self-created reminders to facilitate scheduling the exercises: they set their alarm, made a note in their diaries or printed the exercise and put it on the kitchen Table. A few women refused to set an alarm because they felt embarrassed when others could hear it.

Barriers to scheduling and performing the exercises were related to personal circumstances. Women mentioned the following tasks that conflicted with prioritizing PFMT: having a daytime job, caring for children or relatives, suffering from co-morbidities or just busy with other things, which frustrated them because it made them forget to do their PFMT. Several women said they had a busy job or had children at home and felt particularly unable to create a moment for themselves to practice during the daytime (Q3). Some requirements of the task, consequently, could not be met, such as the recommended training frequency and the training position. Especially exercising in a lying position was a problem for women because they lacked a proper place to practice at work and did not want others to see them practice (Q4).

Personality traits, especially self-discipline, were regarded by women as requirements for fulfilling the training program. Most women said that if they made up their mind to start something, they were determined to finish it: it was part of their character (Q5). They regarded self-discipline as a requirement because no one checked on their progression but themselves. Lack of self-discipline prevented a few women from adhering to the PFMT regime. Women considered themselves as perfectionists, or curiosity made them want to do the exercises correctly and eager to learn about incontinence and the pelvic floor muscles.

Motivation to keep on practicing interacted with the women’s ability to execute the PFMT program. The majority of women perceived the exercises as being not difficult in themselves and easy to perform, but several women felt insecure about the correct contraction or relaxation of the pelvic floor muscles, which made this a barrier to performing the task (Q6). A change in training position could make it even more difficult for them. Tips to check whether they contracted correctly, by vaginal palpation, for example, were helpful to some women but not to all.

Having pre-existing knowledge of how to contract the pelvic floor muscles facilitated the performance of PFMT. This was mentioned by several women who went to see a physiotherapist previously and by women who gained this knowledge from their own profession or from sports experiences, e.g., Pilates (Q7). A few of these women said that it was an advantage of e-Health that they learned more details.

Furthermore, the motivation to adhere to the training program depended on incontinence burden according to most women. Women who experienced incontinence as a heavy burden were strongly motivated to practice, but they were less eager if it was not quite so bothersome(Q8). The progression women made during the training program could be either a facilitator or a barrier: most women who noticed a reduction of urinary leakage or who gained control over their pelvic floor muscles were stimulated to continue because it gave them trust and made them realize that repetition of exercises was essential for a good result and to prevent relapse (Q9). For a few women, however, progression meant a license to slow down the training frequency. Women who made no progression were demotivated or even dropped out of the training program.

### Interaction between PFMT exercises and e-health

The interaction between the PFMT exercises and the e-Health intervention was clearly illustrated by using a stepwise set-up on the website, with every other step providing a more complex exercise. This set-up was perceived by women as a facilitator because the difficulty level of the exercises increased step-by-step, which made them feel guided throughout the program (Q1). Several women appreciated that the first step was an easy exercise, which enabled them to gain trust in their ability to do these (Q2). The stepwise approach made a few women curious about what the next step would bring, making it easier for them to adhere to the training regime (Q3). One woman was aggravated by the fact that not all steps were shown at the start of the program as she preferred to have an overview.

Women mentioned various factors that facilitated the performance of PFMT while using the website. Many women mentioned that the exercises were clearly explained and easy to perform. The language was easy to understand, and the audio-function clarified the text. The images on the webpages supported the text, although a few women preferred to receive more explanation in detailed images of the pelvic anatomy, for example, or in a video (Q4).

Women who were not sure whether they were doing exercises correctly were reassured either by the text (Q5) or by doing the test exercise at the end of each step, which enabled them to check whether they had trained correctly. A few women said that gaining results told them they were on the right track (Q6).

Another website functionality meant that women could follow the steps at their own pace, which was appreciated by several women. Taking more time for a particular step gave them the opportunity to improve rather than to rush through, or to get used to the exercise (Q7).

Within each step, women had to perform the exercises for several minutes, and they were requested to complete a training report in order to continue to the next step. However, a few women did not register these numbers exactly, thus failing to fulfill their training report obligations. They also struggled with timing the length of each exercise and missed a timer function on the website (Q8). They suggested converting the website into a mobile app, which would make it easier to integrate a timer and also to send push messages that could remind them to practice.

The women had access to the e-Health intervention for a total of 3 months. For several women, this period was too long, which made them feel that the length of this training program was not feasible. For others, 3 months was too short, and they lost their motivation to continue practicing because they had not finished yet (Q9). One woman would have liked to receive “maintenance exercises” at the end of the intervention.

## Discussion

### Principal results

This study described a systematic, in-depth analysis of the barriers and facilitators to adoption of a web-based intervention for stress urinary incontinence. We showed that the adoption of e-Health with PFMT among women with SUI mainly depends on the interaction between users and e-Health and users and PFMT exercises. The preference for an accessible self-management therapy facilitated the adoption of a standalone e-Health intervention, which required that women possessed self-efficacy to do this independently. Moreover, they need to be able to schedule the exercises as part of their daily routine and it required the self-discipline to adhere to the training program. Barriers to adopting e-Health were personal circumstances restricting time for PFMT exercises, and feelings of insecurity about performing PFMT due to insufficient skills. Women who felt insecure emphasized they needed personal contact for feedback.

### Comparison with prior work

Our findings are in line with results from previous qualitative studies on the evaluation of digital interventions for urinary incontinence [18–20], despite this qualitative study was conducted in a different setting and country. All studies showed that women appreciated the existence of an accessible tool that gave them the opportunity to solve the problem themselves. This preference for self-management was expressed by the women’s wish to try e-Health as a first step before consulting their doctor, which Asklund et al. also found in their study with a mobile app [19]. Our participants added avoidance of the medical system or surgery as reasons for self-management.

Our study provided new findings in the field of digital interventions for urinary incontinence by investigating adoption, with guidance of the FITT framework. This knowledge is crucial as adoption is an important phase in the implementation process. This study showed that to women with SUI several factors are important for the adoption of an e-Health intervention, namely self-efficacy and self-discipline. Self-efficacy is required not only at the start but also throughout the intervention. At the start, women need to believe in their ability to follow through without the help of a professional, and during the intervention women need to be capable of organizing and performing PFMT. Women who were able to do so regarded the standalone character of e-Health as an opportunity to manage their own treatment. Self-efficacy as a requirement for the adoption of a digital intervention was also shown by Cho et al., who applied the FITT framework to study an app for medication adherence in HIV patients and found that participants with a strong belief in their ability to improve their general physical health were motivated to use the app [31]. Self-efficacy is not a total new concept in the field of urogynecology research as studies with regular, non-digital, PFMT have shown that it is a known predictor for adherence to PFMT [32–34]. Our study added that for e-Health, self-efficacy is not only necessary for adhering to PFMT, but for the adoption of the complete intervention. Another important aspect for the adoption of e-Health was self-discipline. Women regarded this trait as a requisite for adhering to PFMT through e-Health. Women who said to lack this trait needed encouragement from a professional. In implementation research, this is known as “moral obligation”, meaning that people like to be seen as “good” by their clinician to stimulate intervention adherence [35].

Self-efficacy and adherence to PFMT, moreover, depended on women’s skills or previous experiences with PFMT. Gaining physical skill is a known modifier for adherence to PFMT, and Hay-Smith et al. reported that “performing PFMT required a ‘bodily’ knowledge of the pelvic floor muscles that is not easy to achieve” [26]. A subgroup of our participants did indeed struggle with performing the exercises, and this, therefore, underlined the need for personal contact with a professional. For these women the standalone character of the e-Health intervention was a barrier to adopt it. The wish for contact is in line with previous studies [15, 19, 20] although Asklund et al. found a lower need in women using a mobile app, reflected by their overarching theme entitled ‘Enabling my independence’ [19].

Another finding of our study was the importance of the participants’ personal context for adopting the e-Health intervention. Women’s personal circumstances determined not only whether they opted for e-Health but also whether they were able to schedule PFMT. It is known that, in regular, non-digital PFMT, planning, attention and prioritization are modifiers for PFMT adherence [26]. Women have to balance doing PFMT against their other demands in life. In the field of implementation research too, it has been extensively reported that the adoption of self-management solutions was influenced by their alignment with existing routines and practices [35].

The interaction between PFMT exercises and the e-Health intervention appeared to be less relevant for the adoption of e-Health, although some features were explicitly valued by the women. First, the stepwise set-up was appreciated as it guided them through their training program. It is noteworthy that this feature was not mentioned by the women who participated in the Swedish RCT on the effectiveness of the intervention and on which our study was based [18]. Secondly, women found the use of reminder e-mails encouraging to adhere to PFMT, but they would prefer to set their own reminders, which would be facilitated more easily by a mobile app than by a website. Other studies also showed that it is important to set reminders yourself, creating the possibility to engage with the intervention [19, 31].

There is room for improvement in adopting the e-Health intervention. Not all features available in mobile technologies for conservative self-management of urinary incontinence were used optimally [36], such as reinforcements, educational features and possibilities for self-monitoring. It is also remarkable that no mobile technologies tapped into social media resources, although women in this study said they would like to be in touch with peers. This might be due to the assumption that women do not want to disclose their identities out of shame, even if women with incontinence tend to share their information on online platforms anonymously, which illustrates their need for contact with other women [2].

### Future research and implications for practice

There is a great need for online therapeutic interventions for urinary incontinence, especially as only a minority of women consult a health professional [37, 38]. This study showed that e-Health with PFMT is adopted by women with a preference for self-management, who are willing to try this option as a first step before consulting their doctor. A standalone intervention, however, prevents women from being able to ask questions about their progression or about their self-assessed diagnosis. It is important to address this topic in future implementation studies, to make sure women have no remaining complaints or questions. Here one might think of improving technological features to support women in performing PFMT [19] or of incorporating modes of digital communication during the intervention. With regard to this latter option, the question remains if the professionals in primary care should be responsible for the uptake of e-Health in their practice. Previous work showed that while GPs support new digital interventions, they also have mixed feelings about their integration into their daily practice, mostly for practical reasons [14, 39]. To further support the implementation of e-Health for women with SUI, therefore, its feasibility needs to be systematically evaluated, and technical improvements should be made to the intervention when required and available.

### Strengths and limitations

Most studies on the adoption of self-management interventions are designed for patients with known, medically-treated chronical conditions [35], which is different from the perspective of our study. Women with SUI often go undiagnosed, as the condition they suffer from is surrounded by taboo and shame [10]. This study, therefore, adds to the field of implementation research. Another strength is that our e-Health intervention is based on well-developed and evaluated evidence-based intervention [16]. The use of the FITT framework enabled us to provide a more than average in-depth analysis of its adoption, since it provides a tool that encompasses all barriers and facilitators, with the addition of studying the interactions. The limitation of using a framework, however, is that not all data fit into it perfectly. The category of “reminders”, for example, appeared to have an interaction between users and e-Health intervention, but it might also have fitted into the interaction between PFMT exercises and e-Health intervention, as the reminders support the performance of exercises. Another limitation is that recall bias might have occurred, as some interviews took place 6 months after the intervention stopped. During these interviews, we noticed that while these women could not recall some details of the website, they were very well able to undisclosed their personal experiences with the e-Health intervention. Lastly, though we strived to include women with a variety of characteristics, the majority of our participants were highly educated, which might be related to the tendency for e-Health or apps to be more commonly used by people with higher levels of literacy [40]. Another hypothesis is that these women were more prone to participate in research. Qualitative research is by nature not generalizable to the whole population, here women with SUI, as one strives to explore a broad range of opinions around a topic without quantification. Our study reached saturation meaning that no new finding emerged after this amount of participants have been interviewed. However, given the limited diversity in education in this study sample, it would be interesting to investigate the adoption of e-Health for women with SUI with a quantitative design in which other recruitment strategies will be used to ensure representativeness.

## Conclusions

We showed that for stress urinary incontinence, which is often not presented in medical care, e-Health is an appropriate option for a target audience. Use of the FITT framework clearly demonstrates the conditions for optimal adoption. For participants it was a suitable alternative for medical care in person. For others it identified the need for further support by a health care professional. This support could be provided by improvements of technical features and incorporating modes for digital communication. The additional value of integration of the e-Health intervention in primary care might be logical next step and future implementation research needs to focus on feasibility.

## Supplementary Information

**Additional file 1.**

**Additional file 2.**

**Additional file 3.**

## Data Availability

Data that are generated or analysed during this study are included in this published article. Additional file 2 shows the process of analyzing from codes to categories, from categories to themes and interactions of the FITT framework. To guarantee comprehensibility the full list of codes is not included in this file, but is available from the corresponding author on reasonable request.

## Abbreviations

e-Health: electronic health
FITT: Fit between Individuals, Task and Technology
GP: General practitioner
PFMT: Pelvic floor muscle training
SUI: Stress urinary incontinence
