# Supplementary material for: Women’s adoption of a web-based intervention for stress urinary incontinence: a qualitative study
Source: BMC Health Serv Res. 2021 Jun 12;21:574. doi: 10.1186/s12913-021-06585-z (PMC8199839; doi:10.1186/s12913-021-06585-z)
Supplement: Supplementary file 1 — Additional file 1. [file 12913_2021_6585_MOESM1_ESM.docx]

# Interview guide

| **Component of FITT framework** | **Attributes** | **Example questions** |
| --- | --- | --- |
| Individual | Reason for participation | *What induced you to participate? What aspects of this online training program attracted you?* |
|  | Previous experiences with help-seeking, receiving treatment | *Have you sought help for urinary incontinence before, or do you have experience with previous treatment?* |
|  | Expectation of the intervention | *What were your expectations of the intervention?* |
|  | Effects of the intervention on symptoms | *What were the effects of the intervention on your complaints?* |
| Task | Feelings about PFMT | *How were you feeling about PFMT (physically)?* |
|  | Cognitive analysis, planning and attention | *How did you remind yourself to do the exercises?* |
|  | Prioritization | *How did you schedule the exercises? What made it easy or hard to do so?* |
|  | Experiences with adherence | *Could you tell us something about your motivation during the training?* |
|  | PFMT complexity | *Did you find it easy to perform the exercises?* |
|  | Training frequency | *What did you think of the training frequency? How did it fit into you daily life?* |
|  | Effects of the training | *How did the effects of the training influence your motivation?* |
| Technology | Appearance | *What did you think of the website? What could have been better and what should remain? Would you recommend this website to others?* |
|  | Burden of using the website | *How easy was it to use the website to perform the training? What could have been better?* |
|  | Content | *Was it easy to understand the exercises?* |
|  | Stability of website and technical problems | *Did you encounter any technical problems while using the intervention?* |
|  | Attitudes towards the absence of personal contact | *How did you feel about doing this training on your own, without personal contact?* |
|  | Reminders | *What did you think of the e-mail reminders?* |
