# Supplementary material for: Women’s adoption of a web-based intervention for stress urinary incontinence: a qualitative study
Source: BMC Health Serv Res. 2021 Jun 12;21:574. doi: 10.1186/s12913-021-06585-z (PMC8199839; doi:10.1186/s12913-021-06585-z)
Supplement: Supplementary file 2 — Additional file 2. [file 12913_2021_6585_MOESM2_ESM.docx]

## Analyzing from categories to interactions

| **Code name examples** | **FITT Label** | **Category** | **Themes** |
| --- | --- | --- | --- |
| ***Interaction between individuals and technology*** | | | |
| - Appreciated the possibility to ask questions by e-mail | Tech | Absence of face-to-face contact | Self-management / self-efficacy – standalone treatment |
| - Having no face-to-face contact is a challenge | Ind |  |  |
| - Lacking computer skills hampers website use | Ind | Use | Computer skills, co-morbidity - usability |
| - Easy to use on a tablet | Tech |  |  |
| - Free-of-charge intervention | Tech | Accessibility | Personal context – low-key and accessible |
| - Appreciates the anonymity | Ind |  |  |
| - Reminders cause feelings of guilt for non-performance | Ind | Reminder | Feeling supported – reminders |
| - Reminder emails are an incentive | Tech |  |  |
| - Reason for participation is to avoid seeing doctor | Ind | Try as first step | Self-management / self-efficacy – standalone treatment |
| - Reason for participation is self-management of the problem | Tech |  |  |
| - Read-aloud feature is experienced as useful | Ind | Design | Computer skills, co-morbidity - usability |
| - Inconvenient to have many pages for each step | Tech |  |  |
| - Reliability of an e-Health intervention launched by a medical center 1 code | Tech | Reliability | Reliable feeling – fostered by university hospital |
| ***Interaction between individuals and task*** | | | |
| - Knowledge of PFMT because of profession | Ind | Competence | Skills / experience with PFMT - complexity |
| - PFMT familiar because of experience with sports | Task |  |  |
| - Scheduling provides the step with a deadline | Task | Goal setting | Personal circumstances /prioritizing / goal setting / daily routine – scheduling |
| - Motivation: better to do something than nothing |  |  |  |
| - Training adherence successful; stopped using incontinence pads | Ind | Effect on incontinence | Incontinence severity - adherence |
| - Discontinued training program due to lack of progression | Task |  |  |
| - Pelvic floor muscle therapist explains what you need to feel | Task | Experiences from previous treatment | Skills / experience with PFMT - complexity |
| - Previously visited pelvic floor muscle physiotherapist | Ind |  |  |
| - PFMT fully integrated, which facilitates exercise adherence | Task | Integration | Personal circumstances /prioritizing / goal setting / daily routine – scheduling |
| - Routines build trust | Ind |  |  |
| - Scheduling is difficult due to changes in training position | Task | Scheduling | Personal circumstances /prioritizing / goal setting / daily routine – scheduling |
| - Scheduling is successful with more holiday leisure time | Ind |  |  |
| - Consistent performance is required to prevent lapsing | Task | The power of repetition | Progression - adherence |
| - Consistent training boosts self-confidence | Ind |  |  |
| - Difficulty persevering because she doubts if she is doing it right | Ind | Insecurity about exercise performance | Skills / experience with PFMT – complexity |
| - Hard to translate from paper to the body | Task |  |  |
| - Prefers other people not to see her do the exercises | Ind | Performance of the exercise | Personal circumstances – task requirement  AND  Skills / experience with PFMT – complexity |
| - Difficulty relaxing the pelvic floor muscles | Task |  |  |
| - Wants to do exercises well out of perfectionism | Task | Personality trait | Traits (e.g., self-discipline) – adherence |
| - Persevered because it is in her nature to do so | Ind |  |  |
| ***Interaction between task and technology*** | | | |
| - The explanation of the exercises is clear | Task | Clarity of explanation | Clarity and reassurance - content (audio, image, text, test exercise) |
| - Would have liked videos for clarification | Tech |  |  |
| - Taking more time to do a step boosts feeling of improvement | Task | Stepwise set-up | Increasing complexity -Stepwise set-up |
| - Liked the intervention for its step-wise approach | Tech |  |  |
| - Would have liked a timer to measure exercise duration | Task | Functionalities | Task performance - functionalities (e.g., training report) |
| - Preferred an app | Tech |  |  |
| - Reassuring text if exercise was difficult | Task | Mental support | Clarity and reassurance - content (audio, image, text, test exercise) |
| - Website text provides mental support and boosts confidence | Tech |  |  |
| - Easy to persevere as program takes only three months | Task | Intervention duration | Feasibility - access |
| - Three months is too short for this program | Tech |  |  |
