# Supplementary material for: Women’s adoption of a web-based intervention for stress urinary incontinence: a qualitative study
Source: BMC Health Serv Res. 2021 Jun 12;21:574. doi: 10.1186/s12913-021-06585-z (PMC8199839; doi:10.1186/s12913-021-06585-z)
Supplement: Supplementary file 3 — Additional file 3. [file 12913_2021_6585_MOESM3_ESM.docx]

# COREQ Guidelines

**Domain 1: Personal characteristics**

1. Interviewers: MA, LvD and LF

2. Credentials:

*LF (Lotte Firet), MSc, general practitioner in training*

*DT (Doreth Teunissen), PhD, MD*

*RBK (Rudolf Bertijn Kool), PhD, MD*

*LvD (Lukas van Doorn), MSc*

*MA (Manal Aourag), BSc*

*AL (Antoine Lagro-Janssen), Em. Prof, MD
 PA (Pim Assendelft), Prof, MD*

3. Occupation: occupation of MA and LvD: *medical students*

4. Gender: *MA, LF, DT, AL female; RBK, LvD, PA male.*

5. Experience and training: *The interviewers were trained in the theoretic background of qualitative research methodology, in qualitative interviewing and in data analysis.* *The also received practical training in performing qualitative interviews.*

*.*6. Relationship established: *There was no relationship established prior to study commencement*.

7. Participant knowledge of the interviewer. *The interviewers told the participants about their medical background, the goal of the study and about their being involved in a project on e-Health for women with stress urinary incontinence.*

8. Interviewer characteristics: *See 2, 3, 4 and 7.*

**Domain 2: Study design**

Theoretical framework

9. Methodological orientation and theory: *We used thematic analysis, in which we conducted semi-structured interviews that led to themes that provided an answer to the research question. The FITT framework was a tool to guide our analysis.*Participant selection

10. Sampling. *We selected the participants through purposive sampling from the study by Firet et al.(22).*

11. Method of approach. *Participants were emailed to inform them that the researcher would call them to make an appointment. There was no face-to-face contact.*

12. Sample size. *Twenty participants*

13. Non-participation. *Forty-three women were contacted, 23 of whom did not participate. Seventeen of these did not respond to e-mail or telephone messages; three thought it was useless to participate because they dropped out of an early phase of the intervention; two could not participate because of time-constraints; and one did not want to be audio-recorded. One woman did not want to be interviewed by a male (LvD) and was, therefore, interviewed by a female (MA).*

Setting

14. Setting of data collection. *Interviews took place by telephone.*

15. Presence of non-participants*. Interviews 1-2 were conducted by two researchers (LvD and LF). Only LvD was present during interviews 3-10. Interviews 11-13 were conducted by two researchers (MA and LvD). Only MA was present during interviews 14-20.*

16. Description of sample. *See Table 2.*

Data collection

17. Interview guide. *The interview guide was based on the literature, the FITT framework, input from answers to open-ended questions in questionnaires and on the expertise of the supervising committee. We performed semi-structured interviews. The interview guide was pilot-tested twice and adjusted during the study.*

18. Repeat interviews. *Repeat interviews were not carried out.*

19. Audio/visual recording. *We made use of an audio recorder during the interviews.*

20. Field notes. *Field notes were made during and after interviews.*

21. Duration. *Interviews lasted 34 minutes on average.*

22. Data saturation. *Saturation was reached after nineteen interviews.*

23. Transcripts returned. *Transcripts were not returned to participants.*

**Domain 3: Analysis and findings**

Data analysis

24. Number of data coders. *Two researchers independently coded the transcripts.*25. Description of the coding tree. *We did provide a coding tree. The code list is available from the corresponding author on reasonable request.*

26. Derivation of themes. *Themes were derived from the data by using the FITT framework..*

27. Software. *Atlas.ti version 8.4.15. was used for coding.*

28. Participant checking. *Member checking was performed.*

Reporting

29. Quotations presented. *Yes, quotations are presented, mentioning identifier number, age and symptom severity.*

30. Data and findings consistent. *There was consistency between the data presented and the findings.*

31. Clarity of major themes. *We believe major themes are clearly presented.*

32. Clarity of minor themes. *If there were inconsistencies within themes, we provided nuances within the major themes.*
